# The controversy of sports technology: a systematic review

**DOI:** 10.1186/s40064-015-1331-x

**Published:** 2015-09-18

**Authors:** Bryce Dyer

**Affiliations:** Faculty of Science and Technology, Bournemouth University, Fern Barrow, Poole, BH12 5BB UK

**Keywords:** Sport, Technology, Controversy, Fairness

## Abstract

Changes or introductions of technology or equipment can affect how a sport is played or influence its performances. This article conducted a systematic review of peer-reviewed literature to identify any case studies of controversial events surrounding the implementation or use of sports technology. 56 articles were identified that highlighted 31 different case studies found over a three decade time period. Thematic analysis revealed six distinctive themes when reviewing the articles. Whilst the sport of golf had the highest number of case studies, it was found that a significant level of attention had been recorded in the cases of Oscar Pistorius use of prosthetic legs in athletics and the use of full length swimsuits in swimming. The trend surrounding the number of articles was shown to be initially intermittent but saw a peak period from 2008 to 2010. However, the frequency of such articles since this peak has been more consistent. It is proposed that long periods of time without intervention or resolution by a governing body often increases the peer-reviewed attention paid to such cases in examples such as those identified in this systematic review.

**Electronic supplementary material:**

The online version of this article (doi:10.1186/s40064-015-1331-x) contains supplementary material, which is available to authorized users.

## Background

Whilst in many sports it has been proposed that performance is starting to indicate a reducing or plateaued nature in their rate of improvement, it has been claimed that any significant gains in the future will be as a result of technical innovation (Balmer et al. 2011). For example, in speed skating, it has been claimed that half of the progress of world records to date have been as a result of changes in technology with the other half from real athletic improvement (de Koning 2010). Additionally, if a change in sports technology is implemented, its impact in a sport can often be clearly identified (Haake 2009). The implementation of technology has a significant impact in *cycling*, the 100 m *sprint*, and the *javelin* (Haake 2009) as well as the *pole vault* (Haake 2009; Balmer et al. 2011), *long jump*, *high jump*, *triple jump* (Balmer et al. 2011), *amputee sprinting* (Dyer 2015) and *swimming* (Foster et al. 2012; Stefani 2012). As a result, the innovation, design and application of technology to competitive sport is of paramount importance to athletes looking to optimize their best possible performance in the future. Occasionally though, the introduction of new technology can cause debate or controversy. A summary of the philosophical and hypothetical factors regarding the impact or introduction of a product or technology to a sport was summarized by Dyer (2013, PhD theses) and is reproduced in Table 1.

**Table 1:** Summary of sports technology impact

Criterion	References
Harm or health (to the athlete or others)	Hemphill (2009); Kayser et al. (2007); Miah (2006); Lavin (2003); Loland (2002); Stoll et al. (2002); Bjerklie (1993); Brown (1980)
Un-naturalness	Hemphill (2009; Miah (2006); Loland (2002); Stoll et al. (2002)
Unfair advantage or consideration of fairness	Murray (2010); Hemphill (2009); Carr (2008); Miah (2006); Lavin (2003); Stoll et al. (2002); Loland (2002); Gardner (1989); Brown (1980)
Coercion	Lavin (2003); Gardner (1989)
Safety, and spectator appeal	Gelberg (1998)
Integrity of the game, harm to or advantage over the sport itself, or the ‘spirit of the sport’	James (2010); Miah (2000); Gardner (1989)
Deskilling and reskilling	Miah (2006); Sheridan (2006); Miah (2000); Gardner (1989)
Dehumanisation	Miah (2000)
Cost (or excess cost)	James (2010); Froes (1997)
The internal goods of a sport	Schneider and Butcher (1993)
Equal opportunity or access	Lenk (2007); Gelberg (1998)

This criteria is typically relative in nature meaning that the best ethical outcome cannot always be clearly or robustly defined.

The field of study that surrounds the decision making or debate with respect to the actual acceptability, inclusion, or controversy of sport technology has been termed *performance enhancement* (Loland 2009), *technosport* (Freeman 1991; Rintala 1995) *human enhancement technologies* (James 2010) or *mechanical ergogenics* (Holowchak 2002). In addition, if technology has been employed with questions of unfairness or negativity surrounding its use, this has also been termed *technological doping* (Marcinelli et al. 2012) or *technodoping*, (Wolbring and Tynedal 2013).

No formalized review to date has investigated what case studies exist of contentious or controversial sources of sports technology or equipment. In this paper, a systematic review of peer-reviewed literature is conducted into artifact-based sports technology controversy. This is undertaken to identify the type of technology, the sport where this has been found and to conduct a thematic analysis to ascertain any commonality between them. An understanding of any consistent themes may help sports stakeholders to be aware of any potential issues when assessing any new sports technology that they might be faced with.

## Methods

A systematic review of literature was conducted in March 2015 by the author. The systematic review was completed in accordance with the Preferred Reporting Items for Systematic Review and Meta-Analyses (PRISMA) guidelines (PRISMA 2009). However, this protocol was modified by incorporating the article screening process as each article was identified, rather than as a single solitary stage later on in its process. The search strategy used a series of specific keywords. A primary keyword (keyword 1) was used in direct combination with a second (keyword 2) and third keyword (keyword 3). These three keywords used the AND Boolean algebra denotation. The database search terms are presented in Table 2.

**Table 2:** Systematic search terms

Keyword 1	AND keyword 2	AND keyword 3
Sport	Fairness	
Sport	Technology	Enhancement
Sport	Technology	Fairness
Sport	Technology	Controversy
Sport	Technology	Ethics
Sport	Technology	Innovation
Sport	Performance	Enhancement
Sport	Equipment	Fair
Sport	Equipment	Ethics
Sport	Engineering	Ethics
Sport	Technology	Ban
Sport	Unfair	Advantage

Four inclusion criteria were established to exist as a specification for relevance once any sources were identified. The four criteria were:

- Must be peer reviewed based literature.
- Must specifically mention an artefact-based form of sports technology itself and then insinuate (or infer) some level of contention or controversy surrounding it.
- Must be a technological introduction and not technology introduced later due to a change in athletes technique to perform the sport.
- Must be from English text.

Any account of technology in sport was deemed controversial if there was a suggested or implied dispute (or debate) between any of its sports stakeholders regarding its use or inclusion. Alternatively, any mention of a ban of a particular sports technology was also subsequently included in the review results.

This study focused exclusively on artefact-based technology and did not review chemical technology (such as drugs) or genetic-based technology (such as gene therapy,) which were both decided as being outside the scope of this particular study. In addition, editorials were discounted as it was not always clear whether these had been through the same level of peer review as empirical research articles.

Four relevant bibliographic databases were used for this purpose. These included the Sportdiscus, Science Direct, PsycINFO and Medline bibliographic databases. Furthermore, as per the PRISMA protocol, additional records were identified through other sources. These sources included the use of article reference lists identified through the primary search stage and use of Google Scholar.

Once the duplicates had been removed, the remaining publications were then filtered using the inclusion criteria. Subsequently, 56 publications met these conditions and were included in this review.

## Results

The search process yielded the recorded results as shown in Table 3.

**Table 3:** Systematic review results

Number of articles from first search	4996
Number of articles removed due to article duplication/application of eligibility criteria	4955
Number of eligible articles from first search	42
Number of article identified by other means	14
Total number of eligible articles	56

A tabulated record of each sport and its case study are listed in Table 4.

**Table 4:** Search themes case studies

Sport	Sport technology	Reference
Aeromodelling	Whether such technology is a competitive sport	Norris (2011)
Amateur boxing	Increase in head injuries caused by boxers feeling they have an increased invulnerability	Dyer et al. (2010)
America’s Cup yachting	Hull design	Nafziger (2004)
American football	The replacement of leather headgear by plastic helmets	Miah (2005); Gelberg (1995)
Athletics with a disability (100–400 m running)	Oscar Pistorius prosthetic legs	Baker (2015); Wolbring and Tynedal (2013); Marcellini et al. (2012); Wolbring (2012); Burkett et al. (2011); Scholz et al. (2011); Dyer et al. (2011); Howe (2011); van Hilvoorde and Landeweerd (2008); Dyer et al. (2010); Burkett (2010); Murray (2010); Jones and Wilson (2009); Moses (2009); Lea (2009); Weyand et al. (2009); Zettler (2009); Grabowski et al. (2009); Swartz and Watermeyer (2008); Mokha and Conrey (2007); Hutzler (2008); Edwards (2008); Wolbring (2008)
Athletics with a disability (Long Jump)	Markus Rehm prosthetic leg	Baker (2015)
Cricket	Line call technology	Collins and Evans (2011); Collins (2010); Collins and Evans (2008); Singh (2012)
Cycling	Obree ‘tuck position’ (reskilling based technology)	Trabal (2008)
Cycling	Use of the rear gear derailleur in the Tour de France	Trabal (2008)
Cycling	Obree ‘tuck’ or ‘superman position’ (reskilling based technology)	Miah (2006); Trabal (2008)
Distance running	Chip timing	Sailors (2009)
Fishing	Introduction of the spinning reel	Miah (2006); Hummel and Foster (1986)
Football	Use of goal-line technology/refereeing	Svantesson (2014); Singh (2012); Nlandu (2012); Collins (2010)
Golf	Casey Martin’s use of a golf cart	Baker (2015); Burkett et al. (2011); Dyer et al. (2010); Hutzler (2008); Charlish and Riley (2008); Francis (2005)
Golf	‘U’ grooves in golf club heads	Carr (2008); Miah (2006); Gardner (1989)
Golf	Croquet style putter	Carr (2008)
Golf	‘Polara’ golf ball	Dyer et al. (2011)
Golf	Introduction of rubber cored golf ball	Vamplew (2007)
Golf	Golf ball and arguments between two governing bodies over use of steel shafts in the 1950’s	Vamplew (2007)
Ice hockey	Argued mandatory use of goalie face masks	Bachynski (2012)
Ice skating	Use of ‘Klapskates’	van Hilvoorde et al. (2007)
Marathon running	Use of wheelchairs at Boston Marathon in 1975	Hutzler (2008)
Pole vault	Use of glass fibre for pole	Dyer et al. (2010); VerSteeg (2005)
Pole vault	Digging of hole to plant pole into.	VerSteeg (2005)
Rowing	Movable handles (roller skiff)	Trabal (2008)
Swimming	Full body swimsuits being hydrodynamically superior	Foster et al. (2012); Stefani (2012); Craik (2011); Partridge (2011); James (2010); Burkett (2010); Zettler (2009); Lea (2009); Mountjoy et al. (2009); Magdalinski (2000)
Tennis	Use of ‘Hawk-eye’ line judge technology	Collins and Evans (2008, 2011); Collins (2010)
Tennis	‘Spaghetti strung’ rackets	Dyer et al. (2011); Dyer et al. (2010); Sheridan (2006); Savulescu (2006)
Tennis	Ball size and material composition	Sheridan (2006); Miah (2000)
Various	Use of hypoxic chambers	James (2010); Loland and Caplan (2008); Spriggs (2005); Miah (2006); Miah (2005)
Wrestling	Use of video-taped evidence	Nafziger (2004)

31 different case studies were identified in this systematic review. A high number of publications relating to a single case study were the use of prosthetic limbs by Oscar Pistorius (n = 25) and the use of full body swimsuits in swimming (n = 10). The highest number of case studies attributed to a single sport was golf (n = 6). Eligible publications were within a three decade period from 1986 up to 2015. The time series data of the publication volume and rate is illustrated in Additional file 1: Figure S1.

Additional file 1: Figure S1 shows that there has been only a relatively recent record of articles. The data shows that the volume of such articles demonstrates a surge in publication from 2008 to 2012 with the highest amount recorded in 2008. In addition, whilst the volume of articles seems to be reducing from this peak, the frequency of such articles is now continuing to be seen annually.

## Discussion

The debate surrounding controversy in sports technology was clearly detected by this literature review, but it is a relatively recent interest. Despite this, the philosophical debate surrounding the fairness of performance enhancement through sport technology use actually pre-dates this by approximately two decades (Table 1). In addition, it was noted that there were larger volumes of publication in this area in both 2008–2009 and 2011–2012. The reason for these increases can be attributed specifically to the cases surrounding both Oscar Pistorius use of prosthetic limbs and the use of full length swim suit design. The cause of these may well have been triggered by the close proximity of the Olympic Games (held in both 2008 and 2012) whilst also generating great interest due to the relatively long period of time taken by the governing bodies to ultimately resolve them. For example, full length swimsuits were first debated in 2000, (Magdalinski 2000) but not banned until 2011 (Craik 2011). Likewise, Pistorius’s prostheses first drew attention in 2008, but took until 2012 until he finally used them in both the Olympic and Paralympic Games. These durations have provided a significant length of time and opportunity for researchers to investigate or debate their merits. It should be noted that the merits of non-human based decision making systems is still not resolved in several sports, so it is expected that more publications regarding these will likely be seen in the future.

Golf as a sport had the greatest number of different cases occur within it, and these have taken place over the last 100 years. It is possible that the sports relatively long existence and the variety of the equipment’s performance needs to facilitate the game (both ball and club) have provided plenty of opportunity for controversy. However, some of golfs governing bodies have pre-empted this by requiring any new equipment designs to be proactively submitted to them prior to obtaining any approval to be used in matches (USGA 2011). A proactive approval policy such as this would reduce or minimise any future cases of controversy in other sports and likely minimise extensive debate in peer reviewed publications like those that occurred in both swimming and sport with a disability.

The concept of fairness (or unfairness) due to technological introduction was an overarching major theme in every case study example. However, six consistent minor themes emerged from the case studies. These themes are proposed as:

- The use of assistive technology in able-bodied sport.
- Access and parity of sports equipment.
- The introduction of safety equipment in sport.
- ‘Re-skilling’ a sport through the use of new technology.
- ‘De-skilling’ a sport through the use of new technology.
- Governing body oversight issues.

Each of these themes does not have fixed boundaries and it is possible that each case study may overlap with other emerging themes. However, for the conciseness of this review, each case study was categorised by which theme was felt to be most dominant.

### The use of assistive technology in able-bodied sport

The most prevalent case study identified was that of bi-lateral transtibial amputee Oscar Pistorius (n = 23) who wished to compete against able-bodied athletes using two leg prostheses (Baker 2015, Wolbring and Tynedal 2013; Marcellini et al. 2012; Wolbring 2012; Burkett et al. 2011; Scholz et al. 2011; Dyer et al. 2011; Howe 2011; Dyer et al. 2010; Burkett 2010; Murray 2010; Moses 2009; Lea 2009; Jones and Wilson 2009; Weyand et al. 2009; Zettler 2009; Grabowski et al. 2009; Swartz and Watermeyer 2008; Mokha and Conrey 2007; Hutzler 2008; Edwards 2008; Wolbring 2008; van Hilvoorde and Landeweerd 2008). During 2007, Pistorius signalled his intentions to qualify for both the Beijing 2008 Paralympic (Wolbring and Tynedal 2013; Edwards 2008; Wolbring 2008; Mokha and Conrey 2007) and Olympic Games (Burkett et al. 2011; Burkett 2010; Edwards 2008; Jones and Wilson 2009).

Pistorius uses energy storage and return (ESR) lower-limb prostheses. These products are comprised of curved carbon fibre blades (Marcellini et al. 2012) which can vary in shape and geometry (Scholz et al. 2011) and compress and extend under load (Scholz et al. 2011). These can therefore be treated as a perfect spring and subject to Hooke’s Law (Scholz et al. 2011). Such technology initially saw competitive use at the Paralympic Games in 1988 (Scholz et al. 2011).

It has been proposed that ESR’s may provide an unfair advantage (Baker 2015; Wolbring 2012; Scholz et al. 2011; Dyer et al. 2011; Jones and Wilson 2009; Swartz and Watermeyer 2008; van Hilvoorde and Landeweerd 2008) by either increasing the runners speed (or reducing the energy required to do so) and that their design could be optimised to assist in providing it (Marcellini et al. 2012; Lea 2009). A stakeholder-based assessment has subsequently proposed that ESR technology should only be restorative in nature (Dyer et al. 2010, 2011).

The governing body of able-bodied athletics, the IAAF, commissioned a report in late 2007 to assess the technology (Baker 2015; Marcellini et al. 2012; Jones and Wilson 2009; Lea 2009; Zettler 2009; Moses 2009). The results of the report proposed that the technology provided Pistorius with a mechanical advantage over able bodied athletes of more than 30 %, had a 25 % reduced energy output for maintaining the same speed (Marcellini et al. 2012; Jones and Wilson 2009) and possessed inertial benefits due to the reduced mass of the prostheses (Jones and Wilson 2009). However, this research did not consider the performance of the technology during the many phases of a longer sprint event such as the acceleration from the starting blocks (Wolbring and Tynedal 2013), running the bends, or any other additional disadvantages Pistorius felt he personally had (Jones and Wilson 2009). The net result of the report proposed that ESR’s manifest some advantages and Pistorius was subsequently banned by the IAAF to run in able-bodied events (Zettler 2009; Moses 2009). Pistorius himself then commissioned a counter-study (Marcellini et al. 2012) that ultimately demonstrated that whilst he was mechanically different to able-bodied equivalents, he was physiologically similar. His ban was challenged at the Court of Arbitration for Sport (Marcellini et al. 2012; Jones and Wilson 2009; Moses 2009) and was eventually overturned. Pistorius ultimately then competed at both the London 2012 Paralympic and Olympic Games using ESR technology (Wolbring and Tynedal 2013).

A more recent and similar case to Pistorius was that of Markus Rehm. Rehm is a uni-lateral lower-limb amputee who also wished to compete in able-bodied sport in the long jump event (Baker 2015). The controversy concerned the athlete specifically launching himself by using his prosthesis rather than his biological limb. The German Athletics Association considered his prosthetic limb an unfair advantage and would not allow him to participate (Baker 2015). The exact nature of this advantage has not been disclosed, nor investigated in peer reviewed literature to date.

Another prominent case identified was that of Casey Martin (Baker 2015; Burkett et al. 2011; Dyer et al. 2010; Hutzler 2008; Francis 2005). Martin was a professional golfer and a registered disabled citizen who suffered from a circulatory disorder in his lower right leg (Baker 2015; Charlish and Riley 2008; Hutzler 2008) known as Klippel-Trenaunay-Webber syndrome (Francis 2005). Whilst attempting to qualify for the Professional Golf Association (PGA) tour, Martin played golf using a powered golf cart (Hutzler 2008; Francis 2005). He attempted to use this technology to support his transit between strokes (Burkett et al. 2011) but the PGA attempted to prevent this (Francis 2005). Golf carts were banned in professional golf at the time as it was felt that such technology would change the nature of the game by reducing the impact of the walk between each hole (Charlish and Riley 2008) and provide players using them with an advantage over other golfers (Baker 2015; Burkett et al. 2011). Martin attempted to challenge the PGA decision using the American legal system (Francis 2005) by proposing that such technology was part of his professional occupation (Burkett et al. 2011). The Supreme Court ruled that use of the cart would not be a fundamental alteration of the game (Francis 2005), was not considered part of the sport and therefore would not disadvantage other players and organisers (Hutzler 2008). The Supreme Court ultimately ruled in Martin’s favour in 1998 (Silvers and Wasserman 2000).

This wasn’t the first time that the specific needs of the disabled were called into question. There were safety concerns regarding the use of wheelchairs at the Boston Marathon which led to them being outlawed in 1975 (Hutzler 2008).

Whilst these case studies have generally been legally resolved, there still remains a lack of understanding of the role of prosthetic equipment use in able-bodied sport.

### Access and parity of sports equipment

The most cited case study of athlete access to sports equipment surrounds the use of full body swimsuits (n = 10). For the 1992 Olympic Games, Speedo introduced the S2000 fabric (Craik 2011) which reduced the drag of the swimmer by 15 % over more traditional fabrics (Stefani 2012). Speedo’s new fabric ultimately led to them introducing full length swim wear in 1999 (Craik 2011) and at the Sydney 2000 Olympics (Zettler 2009; Magdalinski 2000). In 2000, a media frenzy occurred when it was revealed that the ‘fastskin’ swimsuits would be only given to athletes sponsored by Speedo. These suits were described as a further technological advancement of the company’s ‘aquablade’ swimwear launched prior to the 1996 Olympic Games which had been approved by the governing body FINA (Foster et al. 2012; Craik 2011; Magdalinski 2000). In 2000, the Australian Olympic Committee (AOC) wrote to the Court of Arbitration for Sport (CAS) seeking a ruling on the technologies legality (Magdalinski 2000) which supported that the suits would remain in service. The overarching controversy surrounding of the suit was whether such technology was *fair* (Craik 2011; James 2010; Magdalinski 2000; Partridge 2011; Zettler 2009) *accessible* (Magdalinski 2000) *unfair (*due to its sponsorship limitations) (Partridge 2011; Zettler 2009) or *too expensive* (Zettler 2009). In addition, it was suggested the suits could trap air (thereby improving their buoyancy) and was ultimately a ‘technical aid’ (Baker 2015). Furthermore, later designs such as the ‘X-Glide’ by Arena and the ‘LZR’ by Speedo reduced the cross sectional area of swimmer and subsequently lowered the drag coefficient (Foster et al. 2012; Zettler 2009; Mountjoy et al. 2009; Lea 2009). Finally, it was also claimed that the suits compressed muscles and reduced muscle vibration (Craik 2011), therefore improving endurance performance through the facilitation of venous return (Mountjoy et al. 2009). However, a defence was provided by Speedo that suggested the suit only improved the management of existing forces rather generating new ones (Craik 2011).

The introduction of the Speedo suit in 2000 was shown to improve a swimmers performance by 0-9-1.4 % and further developments yielded a further 1.5–3 % in 2008 (Foster et al. 2012). However, this has been countered by suggesting that the recent return to more basic swimsuit designs has proved no handicap at all (Stefani 2012) and therefore that the full suits proposed benefits might actually be fundamentally psychological in nature (Zettler 2009).

After 43 records were broken in 40 events at the 2009 World Swimming Championships (Partridge 2011; Burkett 2011; Mountjoy et al. 2009) and 130 World Records were broken in less than a year, FINA’s congress finally voted to ban the full length suits (Craik 2011; James 2010) to take effect from Jan 1st 2010 (Foster et al. 2012; Craik 2011). However, any previously established records were still allowed to stand (Baker 2015).

### The introduction of safety equipment in sport

The impact of the *call for* or the *introduction of* safety equipment has occurred in three case studies identified in this review.

Controversy regarding American Football headgear surrounded the introduction of plastic-based helmets in 1939 (Gelberg 1995) which were designed to replace the leather headgear that athletes had previously worn (Miah 2005). The new plastic helmets were lighter, stronger and did not require re-branding after each game (Gelberg 1995). In was envisaged that the adoption of more effective materials and design would reduce head related injuries to players (Miah 2005). However, whilst the number of head injuries has been suggested as decreasing, the severity of those that did occur had been proposed to actually increase (Miah 2005). The suggested reasoning behind this is that players would use the helmet itself as an instrument to perform harder tackles (Miah 2005) or to create a greater sense of invulnerability (Gelberg 1995). Whilst the American judicial system and in its law ultimately clarified liability or blame in any contentious cases involving use of a helmet, it was suggested that potentially dangerous and aggressive tackles have continued regardless (Gelberg 1995). Similar parallels were also evident in amateur boxing whereby controversy was created with their introduction of safety head gear (Dyer et al. 2010). Much like in American Football, the headgear was designed to reduce impact forces but may have indirectly created feelings of athlete invulnerability and therefore encouraged boxers to hit harder or to defend their head less.

Alternatively, despite any safety benefits, discomfort or intimidation has been an alternative problem for headgear adoption. In ice hockey, the adoption of face masks for the goalkeepers or helmets for the field players varied in adoption from the 1950’s up until the end of the 20th century, despite possible face strikes from high speed pucks (Bachynski 2012). In the end, the governing body implemented mandatory use from 1979 (Bachynski 2012).

### The use of non-human decision-making in sport

The key use of non-human decision-making technology has occurred in several sports including *football* (Svantesson 2014; Nlandu 2012; Nafziger 2004), *tennis* (Collins and Evans 2008, 2011; Nafziger 2004), *cricket* (Singh 2012; Collins and Evans 2008, 2011; Nafziger 2004), *rugby*, *golf*, *rowing*, *stock car racing*, *basketball*, *American football* and *wrestling* (Nafziger 2004). It was felt in this review that these could be reviewed under two distinctively different themes. These included *video replay* technology (Svantesson 2014; Collins and Evans 2008; Nafziger 2004) or *line judgement* technology (Svantesson 2014).

#### Video replay technology

Video replay technology has proven controversial in examples such as *golf*, *wrestling*, *rugby*, *American football* and *sculling* (Nafziger 2004).

Criticisms of video-replay technology when used in sport is that the stopping and starting of the game to check the video of a contentious moment will disrupt the flow and pace of football, (Svantesson 2014; Nafziger 2004) cricket (Singh 2012) and in ice hockey (Nafziger 2004). However, in both wrestling and rugby, the refusal by officials to review match footage at key moments then caused controversy which led to legal challenges (Nafziger 2004). Post-game video replay has been successfully implemented feasibly into sport. For example, in 2000, golfer Brian Gay was credited with a birdie shot at the Honda Classic. However, use of video replays after the match indicated that a 16 s delay had taken place between the ball reaching the edge of the hole and then falling in. As a result, the ball was deemed to be ‘out of play’ and a stroke was added to his score (Nafziger 2004).

However, the use of such systems (either in real time or after the sport has concluded) does not ensure a clear decision or outcome. For example, a dispute between two sculls at the finish line of a women’s skull race at the 2000 Olympic Games was challenged when it was deemed that video replay technology did not have the accuracy to judge a difference between two boats separated by 12 one thousandths of a second and therefore relied on human-based judgement (Nafziger 2004).

However, the effectiveness of the official’s decisions without use of video replay has been credited. For example, when a pilot programme was formally implemented in collegiate American football to check judgement call decision making, whilst only 50 of 11,000 games were actually reviewable, only half would have been overturned. This equated to a proportion of incorrect calls of less than a quarter of one percent (Nafziger 2004). Therefore, if human-based decision-making is robustly made, the philosophical debate surrounding line judgement technology is not really a polarised outcome of whether it should be used at all but whether it should act as a decision-maker or merely a decision aid (Collins and Evans 2008).

#### Line judgement technology

In football there has been some controversial decisions made by a human referee with respect to awarding a free kick (Svantesson 2014), giving a penalty (Svantesson 2014) or knowing whether the ball has crossed the goal line (Svantesson 2014; Singh 2012). It is argued that such controversies could have been averted using line judgement technology.

Criticism of line judgement technology has been proposed as dehumanising football (Svantesson 2014), are too expensive (Svantesson 2014, Singh 2012; Nafziger 2004) or impractical to implement at all levels of the football game (Svantesson 2014; Singh 2012). The value of this technology has been disputed by arguing that human mistakes are a facet of both sport and everyday life and that the role of the referee is intended to be based on their interpretation (Nlandu 2012), discretion and instincts rather than just the outright objectivity of the facts (Svantesson 2014). Goal-line technology was fully adopted at the 2014 FIFA World Cup (Svantesson 2014) but it is still not currently used for typical domestic level league competition.

In cricket, controversy has surrounded bowling an illegal delivery of the ball or detecting a ‘leg before wicket’ infringement. This has been attempted to be resolved using line judgement technology such as the ‘Hawkeye’ system (Collins and Evans 2008, 2011). However, controversies over line judgements have occurred when using ‘Hawkeye’ in Tennis, whereby the technology itself may have got a decision wrong (Collins and Evans 2008). This has occurred in a Wimbledon men’s singles final (Collins and Evans 2008) whereby the system judged the ball the wrong side of the line. As per any other form of measurement technology, the ‘Hawkeye’ system does carry a margin of error. It has been proposed that it carries an average degree of accuracy of 3.6 mm in Tennis (Collins and Evans 2008, 2011) or degree of error of 2.6 mm in Cricket (Collins and Evans 2011). Such errors will vary based on ball speed, size of the playing area and recording rates, (Collins and Evans 2011) but there are concerns that without knowing this, naïve spectators might overestimate the technology’s ability to resolve disagreements (Collins and Evans 2008).

### ‘Re-skilling’ a sport through the use of new technology

Reskilling has been proposed to be an increased level of skill required to perform a given task or a substantive change to the skillset (Sheridan 2006).

Technique-based re-skilling was found in golf (Carr 2008) and a croquet style putter design entered use which required a slightly different technique. Golfs governing body banned it as they felt the skill required to use the putter deviated too far from what was traditionally expected in the game of golf (Carr 2008).

Speed skating saw issues regarding the introduction of the ‘Klapskate’ ice skate design in the late 1990’s (van Hilvoorde et al. 2007). This involved an ice skating boot that had a blade that was hinged at its front. This allows the foot to rotate whilst the blade remains in contact with the ice fractionally longer when skating and therefore allows the skater to obtain a longer duration of push (van Hilvoorde et al. 2007). The skate design first entered competitive use in 1997 and required some degree of re-skilling based upon the change in technique required to use it (van Hilvoorde et al. 2007). Not all athletes could initially either access this technology or access effective versions of it (van Hilvoorde et al. 2007). US Speedskating attempted to ban the innovation during the 1997–1998 season, citing that it was an unfair advantage. However other countries objected and the designs originator argued that the skater still had to provide the energy themselves. However he conceded that their use did require a change in skating technique. Whilst all athletes eventually have accessed the technology, some saw their ability within the sport change (positively or negatively) due to their achieved proficiency of the different design (van Hilvoorde et al. 2007).

In tennis, re-skilling-based controversy was generated by the introduction of ‘spaghetti’ strung rackets (Dyer et al. 2010, 2011; Sheridan 2006; Savulescu 2006). Spaghetti stringing was a double layer design of racket strings. These rackets seemed to grab the ball, hold it for fractionally longer and therefore impart excessive spin on the ball (Sheridan 2006; Savulescu 2006) or to generate greater power and accuracy (Dyer et al. 2010, 2011). The controversy for this innovation originated in 1978 and involved opponents becoming confused when receiving a ball delivered from this racket design and led to them making an increasing level of mistakes (Sheridan 2006). The governing body determined that the spaghetti strung racket design compromised the athletic challenge of the game and therefore banned its use (Sheridan 2006).

A similar problem in tennis also surrounded the design of the ball itself. In 1924, despite specifications being known for a balls size, weight and bounce, it was argued that American balls felt lighter in play than European balls (Sheridan 2006). This meant that any players from outside of the balls country of supply would be subjected to a nature of response that they may not have trained for. To address this, the governing body introduced a compression based requirement for balls from 1926 (Sheridan 2006). However, the issue surrounding ball specification arose again in 1999 when tennis’s governing body decided to introduce three new designs of ball onto the male professional circuit, with each tailored to different playing surfaces (Miah 2000). This was due to the premise that tennis had become a game of power serving and that with serves now being in excess of 140 mph, the service speeds were approaching the limits of human reaction time (Miah 2000). Whilst the ethical concerns of adopting these balls were debated (Miah 2000), no resultant outcome was identified in this review.

However, in some cases, a technology which creates some level of re-skilling can be eventually adopted by a sport’s governing body within its constitutive rules. For example, the digging of holes to locate the athlete’s pole in the sport of pole-vaulting was initially considered unethical and subsequently banned by athletics’ governing body. However its structure, dimensions and position was actually later formally adopted by them (VerSteeg 2005). Likewise, the adoption of fibreglass vaulting poles in the 1950’s radically altered the achievable heights by athletes (Dyer et al. 2010) and required athletes to modify their technique (VerSteeg 2005). Fibreglass poles provide an increased mechanical advantage and increased vaulting height to its user by bringing the ends closer together as it bends, thereby temporarily lowering the vaulter’s handgrip. As a result, the pole shortens its overall length by 15–25 % and allows the vaulter to raise their grip in turn by 15–25 % above the height that they are capable of holding without bending the pole (VerSteeg 2005). The new material was initially sanctioned by the international governing body in 1962 but then temporarily banned them in 1972 when it was felt they would have a detrimental impact in the Munich Olympics (VerSteeg 2005). This ban was later overturned. The impact of any governing body changing its mind was also identified in this review. In cycling, Graeme Obree introduced several revolutionary bicycle designs which were raced but then later legislated against (Trabal 2008). However, this example is considered on the boundary of inclusion in this review as it was felt that it was their novel riding position (rather than the actual bicycle), that formed the source of any controversy. One of Obree’s innovations was the ‘superman’ riding position which provided a more aerodynamically efficient method of cycling (Miah 2006).

Likewise, this review detected that the introduction of movable handles on roller-skiffs in some way was contentious to the governing body and reskilled the technique of sculling or rowing (Trabal 2008). However, no further details of this case were found in this review.

In running, controversy arose over whether a chip-based timing system should be used over that of a traditional starting pistol to record runners times (Sailors 2009). By using the newer timing chip technology, each individual would receive an accurate time when they personally crossed the start and finish line. However, this would mean that the visible finishing order may not then be the true positions that they actually finished in. This example involves some level of reskilling as the lack of true positioning of competitors could alter how a runner would approach their pacing strategy as the visual position of a runner might no longer be accurate. The governing body ultimately decided that the starting pistol would remain as the official time (Sailors 2009). However, this decision was challenged in 2008 at the Chicago Marathon whereby a runner who had started 5 min behind the elite category, actually set the 4th fastest time when going by his chip time. The race organisers refused to award him the 4th place prize money as he did not start with the elites (Sailors 2009). A few weeks later, a similar situation occurred again at the Nike Women’s Marathon in San Francisco whereby a runner who had started in a citizens race (some 20 min after the elites), covered the course 11 min faster than anyone else. Ultimately, the governing body ruled that since these runners were not in the elite starting event, they were in a separate race and as such were therefore not entitled to any prize money (Sailors 2009).

### ‘De-skilling’ a sport through the use of new technology

De-skilling insinuates that a sport is made easier to undertake as a result of the introduction of a technology or product (Sheridan 2006). For example, the sport of aeromodelling has suggested that performance enhancements made to the planes design and controls would reduce the technical skill required to fly the planes and perform complex manoeuvres (Norris 2011). Likewise, innovations such as depth finders, bait casting reels and sonar, has increased anglings popularity but deskilled the requirement of fish detection and landing. This ultimately required the introduction of ‘technologically-designed handicaps’ by the sport’s governing body to ensure fair play (Hummel and Foster 1986).

Golf has also seen several examples of such de-skilling controversy. Controversies included club heads which used ‘U’ (or square) grooves (Carr 2008; Miah 2006). This feature provided a greater accuracy of the stroke (Miah 2006). The PGA outlawed the design after it was felt that such designs reduced the skill required to play the game (Miah 2006).

The golf ball also saw complaints and controversy when it moved from its original gutta percha construction to the more modern rubber core (Vamplew 2007). The newer ball design was able to achieve greater travelling distance, therefore requiring fewer strokes to cover the course. The complaints for the new balls adoption originated from the players themselves who had become skilled in its use (Vamplew 2007). This was a case whereby a governing body formally adopted some level of de-skilling to the game it regulated. Conversely, the attempted introduction of the ‘Polara’ golf ball decades later comprised an optimised dimple pattern on its surface which reduced the balls tendency to hook or slice (Dyer et al. 2011). This innovation was described as benefiting lower skilled players more than those who were technically better at the game and was ultimately banned.

In the end, the weight of widespread use of a new technology in society can ultimately force a ruling body to accept a contentious technology. For example, the rear derailleur used on a bicycle is a mechanism used to change gear by driving a chain across a series of wheel mounted sprockets on a bicycle. This innovation was not allowed into the Tour de France until some 40 years after its invention which was years after its initial introduction into commercial bicycles (Trabal 2008). The races director argued that it was dishonest and unfair and insinuated that possessing multiple gears degraded the challenge of the event (Trabal 2008).

### Governing body oversight issues

In some cases, this review highlighted that a sport technology’s controversy stemmed from an absence of any governing body at all. For example, in the America’s Cup, a large volume of rules but the lack of a specific governing body to apply them objectively has led to various disputes over yacht hull design (Nafziger 2004).

Alternatively, having multiple governing bodies of the same sport which operated in different countries has caused issues whereby an innovation was accepted by one governing body but not by another. This occurred with the initial adoption of steel shafted clubs by golfs American governing body from circa 1922, but not by the original governing body located within the Great Britain (Vamplew 2007).

The final example identified in this theme concerned a training technology that would straddle multiple sports. In 2006, the World Anti-Doping Agency (WADA) initiated a consultancy process about the use of hypoxic environments in sport (James 2010; Loland and Caplan 2008). Separate to this, an investigation was also launched by the Australian Football League (AFL) (Spriggs 2005). Hypoxic environment product systems are typically chamber-based (James 2010) or tent-based (Loland and Caplan 2008) and have been used by athletes since the early 1990’s (Loland and Caplan 2008). These environments are intended to provide the performance benefits of altitude simulation (Miah 2005) by providing nitrogen rich air (Spriggs 2005) which increases red blood cell mass (Loland and Caplan 2008; Miah 2006), maximum oxygen uptake (Loland and Caplan 2008) and thereby improve an athlete’s endurance (Loland and Caplan 2008; Miah 2006). Ultimately, this technology allows an athlete to obtain the benefits of training at altitude without having to move their geographical location (Miah 2005). The Australian Institute of Sport has found that such hypoxic systems can produce on average 0.8 % improvement in endurance (Spriggs 2005). WADA felt that such technology violates its ‘spirit of sport’ (Loland and Caplan 2008). However, it has been argued that the criteria which determine whether a technology is against the ‘spirit of sport’ were generalised and hard to be practically applied (Loland and Caplan 2008). Not only this, it has been considered illogical to ban such technology as they are seem equivalent to other methods such as weight training or heat chambers, let alone that athletes would always have the option to train at a higher altitude instead (Spriggs 2005).

Whilst it was proposed that this technology did breach WADA’s guidelines for human enhancement (James 2010), it was not formally banned. Likewise, the AFL declared that such technology did not breach its rules but did ‘send out the wrong message concerning the image of the game’ (Spriggs 2005).

## Conclusion

Four bibliographic databases were used to systematically identify peer reviewed literature regarding any controversy involving the use of technology or equipment in competitive sport. The application of inclusion criteria to the results produced 56 relevant articles, spread over a three decade period from 1986 to 2005. The greatest areas of interest over this time period were the controversy surrounding Oscar Pistorius prosthetic legs in athletics and the use of full body suits in swimming. The sport of golf produced the most number of different case studies. Whilst the majority of cases identified in this review have now been resolved, the role and impact of technology used by an athlete with a disability remains generally unclear and the debate surrounding non-human decision making systems in sport is on-going.

An analysis of the articles revealed six emerging themes of sports technology controversy. These included issues relating to assistive technology, safety equipment, widespread access and/or parity of equipment, non-human decision-making systems, governing body oversight and the impact of de-skilling and re-skilling of a sport due to the introduction of new technology. The overarching theme that straddled all of these emerging areas was the issue surrounding their fairness.

It is proposed that long periods of time without intervention or resolution by a governing body often increases the attention paid to such cases in examples such as those identified in this systematic review.

## Additional file

**Additional file 1: Figure S1.** Graph of eligible example publication rate.
