# Supplementary figures and images for: The controversy of sports technology: a systematic review
Source: Springerplus. 2015 Sep 18;4:524. doi: 10.1186/s40064-015-1331-x (PMC4575312; doi:10.1186/s40064-015-1331-x)

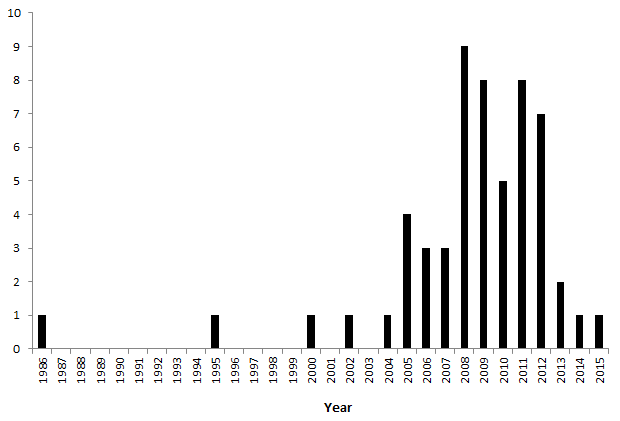


Figure S1. Graph of eligible example publication rate.

Supplement: Supplementary file 1 — Additional file 1: Figure S1. Graph of eligible example publication rate. [file 40064_2015_1331_MOESM1_ESM.docx]
